# Changes in the Expression of Proteins Associated with Neurodegeneration in the Brains of Mice after Infection with Influenza A Virus with Wild Type and Truncated NS1

**DOI:** 10.3390/ijms25052460

**Published:** 2024-02-20

**Authors:** Karin Donátová, Miriam Mladá, Katarína Lopušná, Filip Baran, Tatiana Betáková

**Affiliations:** 1 Department of Microbiology and Virology, Faculty of Natural Sciences, Comenius University in Bratislava, 842 15 Bratislava, Slovakia; donatova3@uniba.sk (K.D.); baran39@uniba.sk (F.B.); 2 Biomedical Research Center, Institute of Virology, Slovak Academy of Sciences, 845 05 Bratislava, Slovakia; miriam.hanckova@savba.sk (M.M.); katarina.lopusna@savba.sk (K.L.)

**Keywords:** influenza virus, adaptation, NS1 protein, brain, immune response, inflammation, neurodegeneration

## Abstract

Influenza type A virus (IAV) infection is a major cause of morbidity and mortality during influenza epidemics. Recently, a specific link between IAV infection and neurodegenerative disease progression has been established. The non-structural NS1 protein of IAV regulates viral replication during infection and antagonizes host antiviral responses, contributing to influenza virulence. In the present study, we have prepared a mouse lung-to-lung adapted to the NS1-truncated virus (NS80ad). Transcriptome analysis of the gene expression in the lungs revealed that infection with wild-type A/WSN/33 (WSN), NS80, and NS80ad viruses resulted in different regulation of genes involved in signaling pathways associated with the cell proliferation, inflammatory response, and development of neurodegenerative diseases. NS1 protein did not influence the genes involved in the RIG-I-like receptor signaling pathway in the brains. Lethal infection with IAVs dysregulated expression of proteins associated with the development of neurodegenerative diseases (CX3CL1/Fractalkine, Coagulation factor III, and CD105/Endoglin, CD54/ICAM-1, insulin-like growth factor-binding protein (IGFBP)-2, IGFBP-5, IGFBP-6, chitinase 3-like 1 (CHI3L1), Myeloperoxidase (MPO), Osteopontin (OPN), cystatin C, and LDL R). Transcription of GATA3 mRNA was decreased, and expression of MPO was inhibited in the brain infected with NS80 and NS80ad viruses. In addition, the truncation of NS1 protein led to reduced expression of IGFBP-2, CHI3L1, MPO, and LDL-R proteins in the brains. Our results indicate that the influenza virus influences the expression of proteins involved in brain function, and this might occur mostly through the NS1 protein. These findings suggest that the abovementioned proteins represent a promising target for the development of potentially effective immunotherapy against neurodegeneration.

## 1. Introduction

Although influenza is considered a respiratory infection, the influenza A virus (IAV) can spread to and replicate in numerous organs such as the heart, thymus, liver, spleen, kidneys, etc. [1]. Recent studies suggest that influenza virus infection is associated with the development of neurological diseases [2,3,4].

Some neurotropic/neurovirulent strains can infect the central nervous system (CNS), resulting in neuronal cell death in the substantia nigra pars compacta and the production of proinflammatory and inflammatory cytokines by glial cells, leading to the development of neuroinflammation [5,6,7]. The immune response can irreversibly disrupt the complex structural and functional architecture of the CNS, frequently leaving the patient with a poor or fatal prognosis. However, non-neurotropic viruses are also involved in the development of neurological complications [8]. Acute influenza-associated encephalopathy/encephalitis (IAE) is one of the rare complications of IAV infection [9]. Encephalopathy may include a variety of clinical–radiological syndromes or acute encephalopathy syndromes. Viruses present in the mucosa of the upper respiratory tract can directly infect olfactory sensory neurons present in the olfactory epithelium. Anterograde axonal transport leads to the dissemination of the virus within axonal bundles passing through the cribriform plate into the olfactory bulb. Trans-synaptic distribution to mitral cells results in virus spread along the olfactory tract to other brain regions [10]. Encephalitis caused by IAV is associated with the development of Parkison’s disease [11]. It was shown that microglia, CNS-resident macrophages, are sensitive to neuroinflammation and inflammatory cytokines and can inhibit neurotropic factor signaling and hippocampal long-term potentiation, which can lead to long-lasting brain dysfunction and increased susceptibility to inflammatory stimuli [12,13,14].

IAV belongs to the family *Orthomyxoviridae*. This genome contains eight segments of single-stranded RNA and encodes up to 18 proteins. The eighth segment encodes a multifunctional non-structural NS1 protein. The NS1 protein regulates viral replication during infection and antagonizes host antiviral responses, contributing to influenza virulence. The N-terminal RNA binding domain of NS1 protein inhibits interferon (IFN) response by recognizing and binding dsRNAs and plays an essential role in the translation of viral mRNA [15]. The short linker region separates the N-terminal and effector domain, which can interact with more than 50 host proteins and has multiple functions. This domain can bind CPSF30 and inhibit the recognition of the CPSF complex of polyadenylation signals at the 3’end of mRNAs during transcription, the cleavage of immature pre-mRNAs, and the recruitment of the poly(A) polymerase to synthesize poly(A) tail [16]. The effector domain prevents activation of the interferon transcription factors (IRF) 3, nuclear factor-kappa beta (NF-κB), cJun/ATF2, and hence, the synthesis of type I interferons and proinflammatory proteins [17,18]. The C-terminal domain of the NS1 protein is associated with the inhibition of IFNs and cytokine production in the infected cells, inhibits apoptosis, and is responsible for the activation of the PI3K pathway [19]. The C-terminal region can modulate pathogenicity through interaction with PDZ-binding protein(s) [20,21]. Mutations and deletions in the NS1 protein are associated with increased IAV virulence [19,22]. NS80 virus, which encodes only the first 80 aa of the NS1 protein, was prepared by a reverse genetic system. This virus replicates in the cells to lower viral titer and induces high levels of proinflammatory cytokines and chemokines, which are associated with increased pathology [19,23].

It is known that the NS1 protein deregulates many host factors. The present study aims to explore the effect of adaptation of NS1 truncated virus (NS80) on the replication of the virus, regulation of intracellular gene expression, and dissemination of the virus into the brain. We have identified NS1-deregulated genes in the lungs of infected mice and the signaling pathways associated with the development of neurodegenerative diseases. NS1 protein affects the expression of the proteins that are responsible for brain function.

## 2. Results

### 2.1. Impact of Adaptation on Replication of NS1 Deletion Mutant

Viruses WSN and NS80 were prepared by reverse genetic system and differ only in deletion in NS1 protein. Even though the NS80 virus was attenuated in the Balb/c mice and was not able to replicate to high titer in the lungs, this virus was more pathogenic than the control WSN virus [24]. To determine whether adaptation of the NS80 virus will result in higher replication and mortality, the NS80 and WSN viruses were passaged through the lungs as described in Materials and Methods. To identify which mutations are associated with the adaptation of WSN and NS80 viruses, we analyzed SNPs from RNA-seq data. Adaptation of the WSN virus resulted in mutation in polymerase basic 2 (PB2) protein (I517V) and M2 protein (R45H). Adaptation of the NS80 virus resulted in mutations in PB2 protein (D87N and I517V), polymerase acid (PA) protein (D87N), and hemagglutinin (HA) (202G).

**Figure 1:**
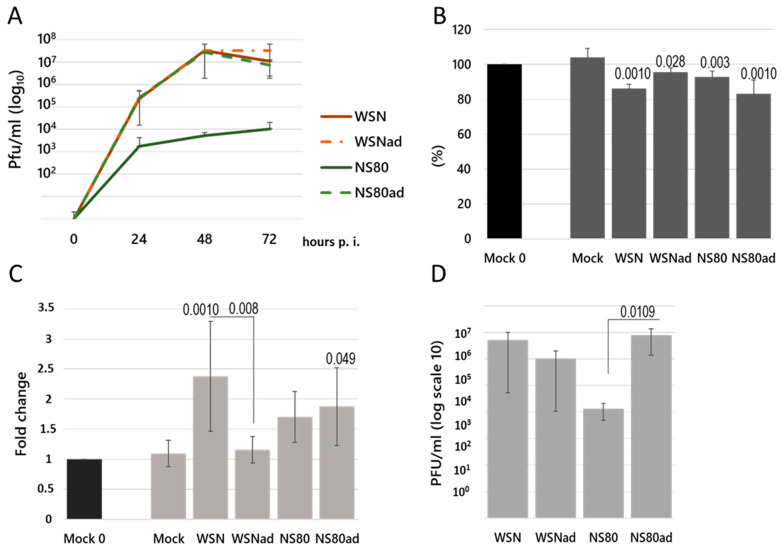
(**A**) Confluent monolayers of Vero cells were infected with viruses at MOI of 0.01 and incubated at 37 °C. At different time points, virus titer in culture medium was determined on Vero cells as described in Materials and Methods. The data shown represent the mean ± SD for three independent experiments. (**B**) Percentage of body weight loss relative to the initial weights (day 0) was recorded on the 3rd day p.i. in each group of mice infected with influenza virus WSN, WSNad, NS80, and NS80ad. The expression values represent the mean of three separate experiments and are presented as the mean ± SD. Data were statistically evaluated using one-way ANOVA and post hoc Tukey’s HSD test; *p* < 0.05; *p* < 0.01; *p* < 0.001. (**C**) Percentage of weight of lungs relative to the initial weights (day 0) was recorded on the 3rd day p.i. Results are presented as mean ± SD (*n* = 3). Statistically significant differences between WSN, WSNad, NS80, and NS80ad are indicated as follows: *p* < 0.05; *p* < 0.01; and *p* < 0.001. (**D**) The virus titer was determined in the lung tissue homogenates as described in Materials and Methods. Results are presented as mean ± SD (*n* = 3). Data were statistically evaluated using one-way ANOVA and post hoc Tukey’s HSD test; *p* < 0.05.

Adapted viruses were propagated in Vero cells, and the virus titer was determined. The Vero cells were infected with non-adapted (WSN and NS80) and adapted (WSNad and NS80ad) viruses at an MOI of 0.01, and the virus titer in the supernatants of infected cells was determined at different times post-infection (Figure 1A). Adaptation of the NS80 virus resulted in increased replication of the NS80ad virus in Vero cells.

Balb/c mice were intranasally infected with non-adapted and adapted viruses (PFU 10^3^), and the lungs were analyzed on the third day post-infection (p.i.). All infected mice showed some signs and symptoms of illness and exhibited weight loss (Figure 1B). Mice infected with NS80 and NS80ad viruses were especially more lethargic and had more bristled fur than mice infected with WSN and WSNad. The weight of the lungs dissected from the mice infected with WSN or NS80ad viruses was significantly higher when compared to WSNad (Figure 1C). Despite the fact that infection with the WSNad virus resulted in macroscopic changes in the lungs similar to the lungs infected with WSN, NS80, and NS80ad viruses, the weights of the WSNad-infected lungs did not significantly increase. On the other hand, the weights of the mice did not decrease as much as in mice infected with WSN, NS80, and NS80ad viruses. The viral titer was determined in the lungs on the 3rd day post-infection. Adaptation of the WSN virus did not result in increased viral replication in the lungs. On the other hand, the NS80 virus was attenuated and did not replicate in the lungs to a high titer. After adaptation, the NS80ad virus replicated in the lungs very well, and the viral titer was similar to the titer of the WSN virus (Figure 1D).

### 2.2. Transcriptome Analysis of the Gene Expression in the Lungs Infected with Non-Adapted and Adapted Viruses

To detect changes in the gene expression after infection with the adapted NS80 virus, we performed RNA sequencing in the lungs infected with non-adapted and adapted viruses. In total, ~126, 129, 39, 126, and 135 million raw sequencing reads were obtained from the mock, WSN, WSNad, NS80, and NS80ad, respectively. After quality analysis of sequencing and filtering, we obtained ~125 million reads in the mock group, ~217 million reads in the WSN infected group, ~40 million reads in the WSNad group, ~126 million reads in the NS80 group, and ~134 million in the NS80ad group (Supplement Table S1). All groups consist of three mice, except for the mock group, which consists of two mice, and WSNad, which represents only one mouse. One sample prepared from a mock mouse and two samples prepared from the WSNad-infected mice did not pass the quality analysis control and were not sequenced. All the reads were mapped to the *Mus musculus* genome GRCm39 by HISAT2 version 2.0.5 software The total mapping ratio was 87.8%, 93.1%, 93.3%, 91.55%, and 92.86% for mock, WSN, WSNad, NS80, and NS80ad groups, respectively, indicating the high quality of the sequencing data.

### 2.3. Truncation of NS1 Resulted in Promotion of Expression of Genes Involved in Cell Proliferation

**Figure 2:**
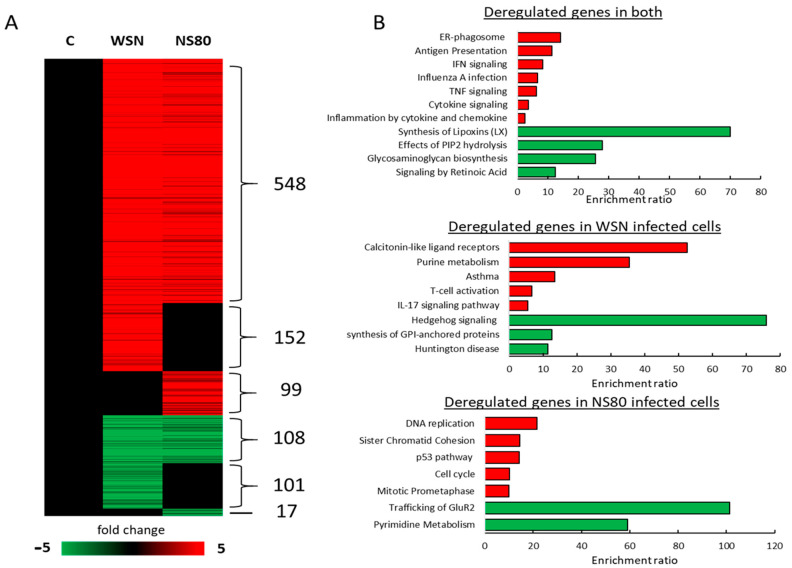
Global gene expression RNA from lungs infected with WSN and NS80 viruses. (**A**) Heatmap showing fold change expression values of differentially expressed genes (FC ≥ 2, *p* < 0.05 by DESeq2) in WSN (n = 3) and NS80 (n = 3) infected lungs when compared to uninfected lungs (C, n = 2). (**B**) KEGG, Panther, and Reactome pathway analysis of genes presented in a. Analysis was performed from genes differentially expressed in both WSN and NS80 infected lungs relative to uninfected lungs (deregulated genes in both); genes differentially expressed only in WSN infected lungs relative to uninfected lungs (deregulated genes in WSN infected cells) and genes differentially expressed only in NS80 infected lungs relative to uninfected lungs (deregulated genes in NS80 infected cells). Red pathways consist of upregulated genes and green pathways of downregulated genes.

To analyze the effects of the NS1 protein and adaptation of the NS80 virus on intracellular gene expression, we performed global gene expression from lungs infected with WSN, NS80, and NS80-adapted viruses. We identified 757 differentially expressed genes (DEGs) (FC > 2, *p* < 0.05) in cells infected with WSN relative to uninfected (NC) cells and 772 DEGs in cells infected with NS80 virus (Figure 2A, Supplementary Figure S1), 548 upregulated and 108 downregulated genes overlapped between WSN and NS80 mutant. The most upregulated genes were related to proinflammatory cytokines (e.g., Cxcl9, Cxcl10, Ccl2), while the most downregulated genes were involved in lipid binding (Crabp1) and cytoskeletal protein binding (Stmnd1, Crabp1). In addition, multiple poorly characterized protein-coding genes, such as Gm25810, Gm24592, Gm25683, and non-coding RNA genes (e.g., Mir1946b), were also deregulated. Common upregulated genes were involved in IFN signaling, Influenza A infection, and inflammation (Figure 2B) since downregulated genes were involved in pathways such as the Synthesis of lipoxins and Effects of PIP2 hydrolysis.

In addition, we identified 152 upregulated and 101 downregulated genes specific to WSN-infected cells (Figure 2A). These genes were involved in signaling pathways such as Calcitronin-like ligand receptors, T-cell activation, IL-17 signaling, Hedgehog signaling, and others (Figure 2B). We also identified 99 upregulated and 17 downregulated genes only in cells infected with the NS80 virus (Figure 2A). These genes were involved mainly in DNA replication, Sister chromatin cohesion, and cell cycle signaling pathways (Figure 2B).

### 2.4. Influenza Viruses Deregulated Genes Involved in the Development of Neurodegenerative Diseases

**Figure 3:**
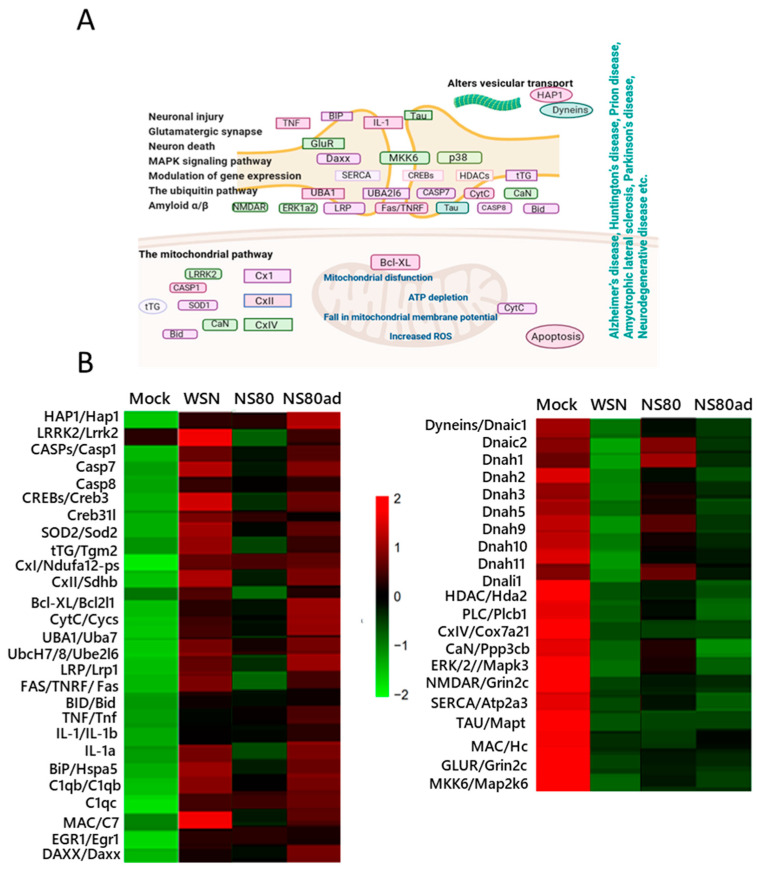
KEGG pathway and GO analysis. (**A**) Schematic representation of key genes involved in the signaling pathways associated with brain damage. Picture was created on BioRender.com, accessed on 15 January 2024. (**B**) Heatmap of averaged log2(FPKM + 1) values of genes enriched in signaling pathways connected with brain damage (from a.) in WSN (n = 3), NS80 (n = 3), and NS80ad (n = 3) infected lungs. Red color indicates genes with high expression levels, and green color indicates genes with low expression levels.

Pathway analysis from data obtained in RNA/seq showed that genes deregulated after influenza infection are involved not only in the influenza virus replication, immune response regulation, etc., but also in regulation of neuronal injury, glutamatergic synapse, neuron death, MAPK signaling pathway, ubiquitin pathway, amyloid α/β, and mitochondrial pathway (Figure 3A). The upregulation or downregulation of these genes is closely associated with the development of Alzheimer’s disease, Huntington’s disease, Prion disease, Amyotrophic lateral sclerosis, Parkinson’s disease, and neurogenerative disease.

To understand the potential function of differently expressed genes, GO analysis was performed. The results showed that the target genes in WSN-infected lungs were mainly associated with hydrolase activity, cytokine and chemokine activity, cellular amino-acids and carbohydrate metabolic process, cell death, and immune response (Figure 3B and Figure S2). On the other hand, infection with the NS80 virus upregulated and downregulated genes is associated with receptor activity, epidemic development, immune response, chemokine, and cytokine activity, etc. Genes differently induced after infection with NS80ad compared to NS80 virus were involved mainly in the regulation of growth, response to biotic stimulus, DNA replication, receptor activity, immune response, etc.

### 2.5. Adaptation of Influenza Viruses with Truncated NS1 Protein Resulted in Induced Expression of Genes Involved in Neuroinflammatory Response in Lungs

**Figure 4:**
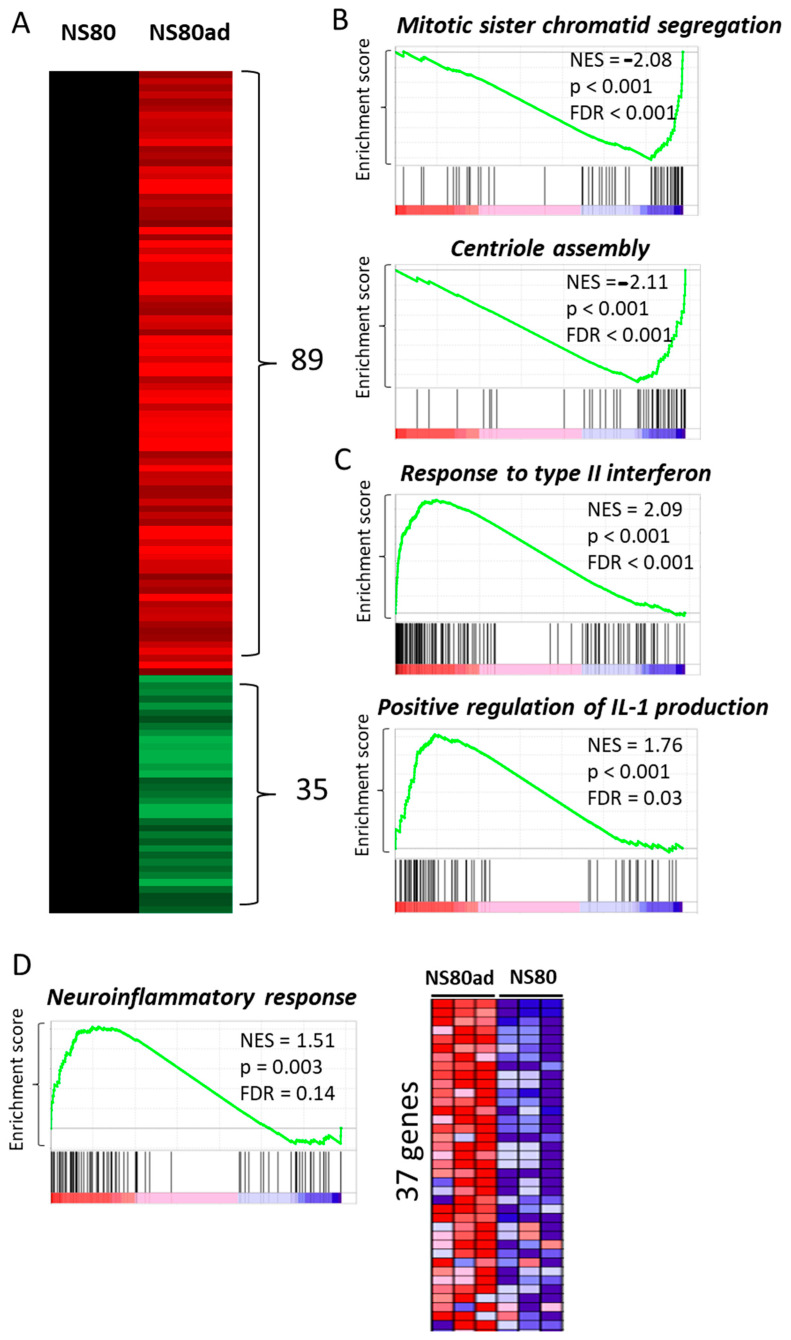
(**A**) Heat map showing fold change expression values of subset of differentially expressed genes (FC ≥ 2, *p* < 0.05 by DESeq2) in NS80ad infected lungs (n = 3) when compared to NS80 infected lungs (n = 3). (**B**) GSEA, using RNA-seq data, shows negative enrichment in Mitotic sister chromatid segregation and Centriole assembly in NS80ad-infected lungs (n = 3) when compared to NS80-infected lungs (n = 3). Normalized enrichment scores (NES), false discovery rate (FDR), and *p*-values are shown. (**C**) GSEA using RNA-seq data shows positive enrichment in response to type II interferon signaling and positive regulation of IL-1 production in NS80ad-infected lungs (n = 3) when compared to NS80-infected lungs (n = 3). Normalized enrichment scores (NES), false discovery rate (FDR), and *p*-values are shown. (**D**) GSEA using RNA-seq data shows positive enrichment in neuroinflammatory response in NS80ad-infected lungs (n = 3) when compared to NS80-infected lungs (n = 3). Normalized enrichment scores (NES), false discovery rate (FDR), and *p*-values are shown. (right) Heatmap showing expression of 37 genes in NS80ad infected lungs (n = 3) and NS80 infected lungs (n = 3) from neuroinflammatory signaling gene set. High expression is indicated by red, while low expression is shown by blue.

Next, we analyzed deregulated genes in lungs infected with the adapted NS80 virus (NS80ad). We identified 89 upregulated and 35 downregulated genes relative to lungs infected with the NS80 virus (Figure 4A). Among the signaling pathways negatively enriched in global gene expression data analysis using GSEA version v3.0 software were, e.g., Mitotic sister chromatid segregation and Centriole assembly (Figure 4B). Among the positively enriched signaling pathways were, e.g., Cellular response to type II interferon and Positive regulation of interleukin production (including interleukin 1, 6, 8, 10, 12, and 13) (Figure 4C). In addition, we observed positive enrichment in neuroinflammatory response (Figure 4D).

It is highly interesting that interferon and interleukin signaling pathways were not positively enriched in the case of comparison of NS80 and NS80ad-infected lungs with WSN-infected lungs. Our results suggest that adapted influenza viruses lacking NS1 protein induce stronger interferon and interleukin responses in infected cells and are capable of inducing strong neuroinflammatory responses in the lungs.

### 2.6. Adaptation of NS80 Virus Increased Pathogenicity of the Virus

**Figure 5:**
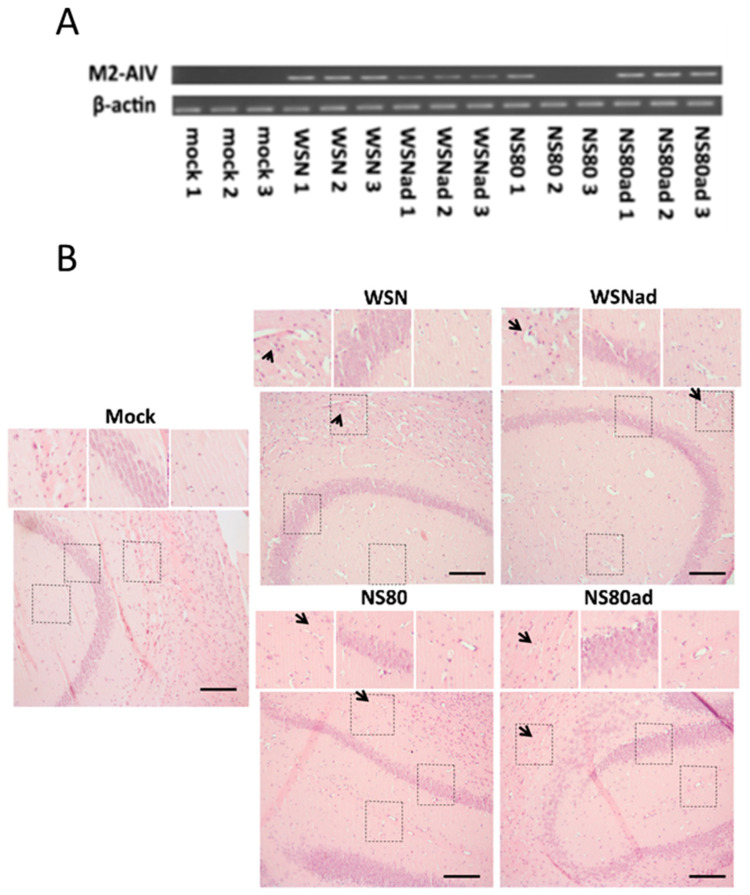
(**A**) Detection of non-adapted and adapted viruses in the brains. Three Balb/c mice were infected with WSN, WSNad, NS80, and NS80ad viruses. The brains were harvested on the 3rd day p.i. Mock represents samples from non-infected brains. RNA was purified from the homogenates, and RT-PCR used primer against M2 protein. (**B**) Histopathology of brain tissues. Mice’s (Balb/c) brain parts were dissected and fixed on the 3rd day p.i. Sections were obtained using a microtome. Hematoxylin and eosin staining were carried out: Mock—non-infected mice; WSN, WSNad, NS80, and ND80ad-infected mice. Insets show higher magnification views of the selected areas. The necrotic lesions are labeled with arrows. Scale bars: 100 µm.

KEGG pathway analysis indicated that adaptation of the NS80 virus might be associated with neurological complications. To find out how the adaptation of the virus influenced the virus’s spread to other organs, especially the brain, we analyzed the brains of mice infected with non-adapted and adapted viruses. The Vero cells were infected with the supernatant from the brain homogenates, and a TCID_50_ assay was performed. None of the tested viruses were detected by this method. Thereby, the presence of the virus was tested by RT-PCR. Viral RNA was detected in the brains infected with WSN, WSNad, and NS80ad virus (Figure 5A). Only one of three samples obtained from the brain infected with the NS80 virus was positive. The obtained results showed that adaptation of the NS80 virus may increase the ability to overcome barriers and spread to other organs.

Hematoxylin and eosin-stained paraffin sections of the uninfected brain are free of necrosis and inflammation. Histopathology changes were observed in the hippocampus after infection with all influenza viruses (Figure 5B). Infection with WSN and WSNad resulted in severe necrotic lesions in the pyramidal cell layer in the hippocampus (arrows). Adaptation of NS80 resulted in increased pathology and larger necrosis than non-adapted NS80.

### 2.7. Activation of Genes Involved in RIG-I like Receptor Signaling Pathway Was Not Affected by NS1 Protein

**Figure 6:**
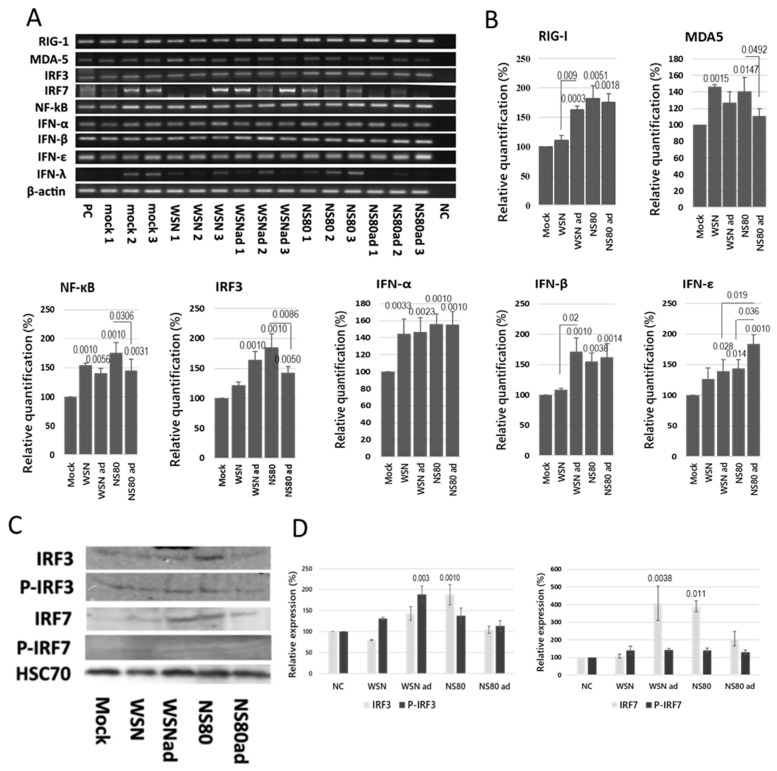
Induction of RIG-I-like receptor signaling pathways in the brains. BALB/c mice were infected intranasally with LD100 doses of WSN, WSNad, NS80, and NS80ad viruses. The brains were harvested from non-infected mice and from infected mice on the 3rd day p.i., and brain homogenates were used for analyses. Representative (**A**) reverse transcription PCR blots and (**B**) relative expression levels of mRNAs were obtained. The expression values represent the mean of three separate brains and are presented as the mean ± SD. Data were statistically evaluated using one-way ANOVA and post hoc Tukey’s HSD test; *p* < 0.05; *p* < 0.01; *p* < 0.001. RIG, retinoic acid-inducible gene; IFN, interferon; IRF, interferon regulatory factor; p.i., post-infection; MDA5, melanoma differentiation-associated gene 5. (**C**) Immunoblot of IRF3, phosphorylated IRF3, IRF7, and phosphorylated IRF7 in the brains of mice infected with WSN, WSNad, NS80, NS80ad, or mock control. HSC70 served as loading control. (**D**) Relative expression levels in Western blot are presented as the mean of three independent experiments and presented as the mean ± SD. Data were statistically evaluated using one-way ANOVA and post hoc Tukey’s HSD test; *p* < 0.05; *p* < 0.01; *p* < 0.001.

The RIG-I/MDA5 signaling pathways are responsible for the activation and production of interferons, cytokines, and chemokines. To find out how the adaptation of the viruses influenced the activation of these signaling pathways, the RNA was isolated from the brains, and transcriptional profiles were investigated on the third day post-infection. The same amount of RNA was used in RT-PCR. The expression of mRNA levels between different samples was normalized by using β-actin as an internal control. All viruses, except WSN, significantly increased expression of RIG-I and IRF3 mRNAs (Figure 6A,B). The level of MDA5 mRNA was significantly increased only in the brains infected with non-adapted viruses. The level of NF-κB and IFN-α mRNAs was significantly increased in all infected brains. The NS80ad virus induced lower levels of IRF3 and NF-κB mRNAs more than the NS80 virus. The IRF7 mRNA was not detected in all samples obtained from the brains infected with WSN and NS80ad viruses. Transcription of IFN-β and IFN-ε in the brains infected with the WSN virus was similar to that of non-infected brains (mock). Transcription of genes involved in RIG-I-like receptor pathways was activated independently on the NS1 protein.

Small amounts of IRF3 and IRF7 proteins were detected in the brain samples by using Western blot. Production of IRF3 protein was significantly increased in the brains infected with the NS80 virus (Figure 6C,D). Phosphorylation of IRF3 was increased in the brains infected with WSNad virus. The expression of IRF7 protein was increased in the brains infected with WSNad and NS80 viruses. IRF7 proteins were phosphorylated, but the level of phosphorylation was not higher in infected brains than in non-infected brains.

### 2.8. Transcription of GATA3 mRNA Was Downregulated in the Brains Infected with NS1 Truncated Viruses

**Figure 7:**
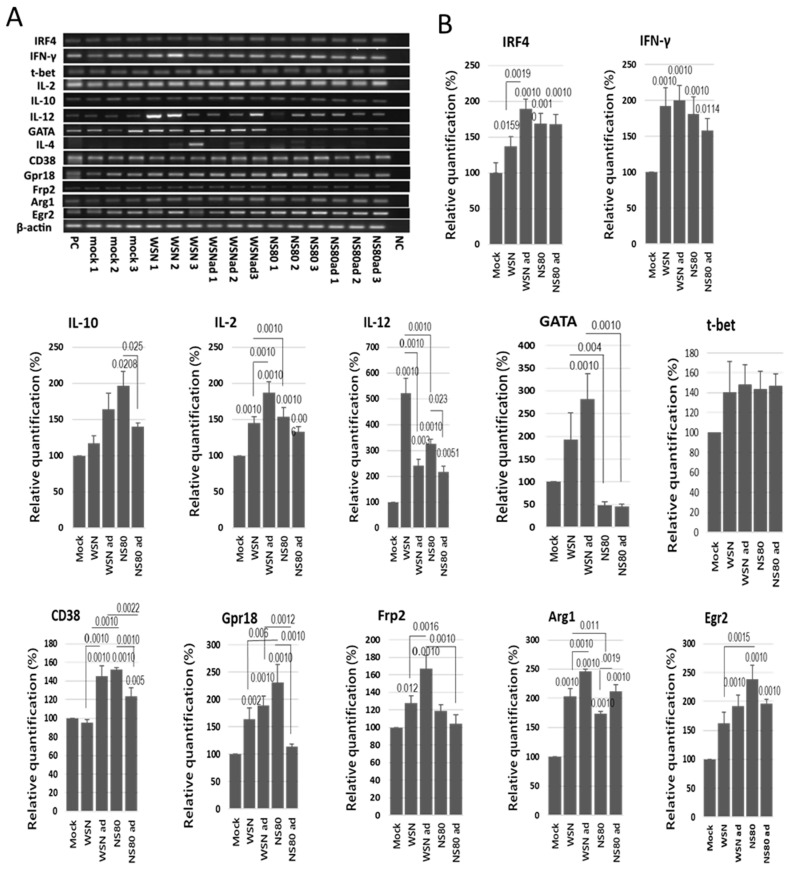
Induction of cytokines, Th, and macrophage markers in the brains. BALB/c mice were infected intranasally with LD100 doses of WSN, WSNad, NS80, and NS80ad viruses. The brains were harvested from non-infected mice and from infected mice on the 3rd day p.i., and brain homogenates were used for assessment of mRNA levels. Representative (**A**) reverse transcription PCR blots and (**B**) relative expression levels of mRNAs were obtained. The expression values represent the mean of three separate brains and are presented as the mean ± SD. Data were statistically evaluated using one-way ANOVA and post hoc Tukey’s HSD test; *p* < 0.05; *p* < 0.01; *p* < 0.001.

The translation factors IRF4, IFN-γ, CD38, Gpr18, Frp2, Arg1, and Egr2 are involved in the regulation of immune response and are essential for M1 and M2 macrophage polarization. T-bet and GATA3 transcription factors orchestrate the Th1 and Th2 differentiation and regulate expression of IL-2, IL-4, IL-10, IL-12, etc. To find out how NS1 protein and adaptation influence the immune response in the brains, the abovementioned transcription factors were investigated. All viruses significantly increased the levels of IRF4, IFN-γ, IL-2, IL-12, CD38, Gpr18, and Arg1 mRNAs (Figure 7A,B). None of the tested viruses induced the transcription factor t-bet. Transcription of IL-10 mRNA was induced only by NS80 and NS80ad viruses. The WSN virus did not influence the transcription of CD38 and Egr2. Transcription of Frp-2 mRNA was increased only in the brains infected with WSN and WSNad viruses. Adapted viruses induced significantly higher levels of Arg-1 than non-adapted viruses. Only WSNad increased the transcription of GATA3 mRNA. It is interesting that both NS80 and NS80ad viruses inhibited the transcription of GATA3. The transcription of GATA3 was not dependent on the adaptation of the NS80 virus but was evidently affected by truncated NS1 protein.

### 2.9. The NS1 Protein Might Regulate Expression of Myeloperoxidase (MPO) Protein in the Brain

**Figure 8:**
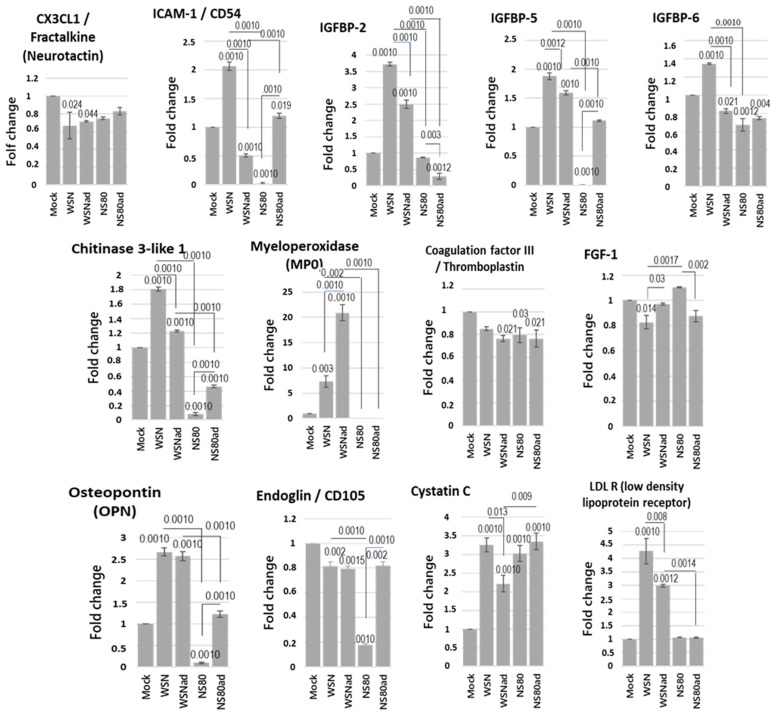
Cytokine expression in the brains of mice infected with LD100 doses of WSN, WSNad, NS80, and NS80ad viruses. Protein expression levels of cytokines in the brain of infected mice harvested on the 3rd day p.i. were determined by cytokine array. The expression levels of cytokines were normalized to the expression level of reference spots. The assay was performed in duplicate to ensure reproducibility of the results. Results are presented as mean ± SD (*n* = 3). Data were statistically evaluated using one-way ANOVA and post hoc Tukey’s HSD test; *p* < 0.05; *p* < 0.01; *p* < 0.001.

Data obtained from the investigation of the transcriptional activity suggest that the immune system in the brain was activated. To detect the cytokines and soluble proteins in the brain, the Proteome Profile mouse XL cytokine assay was performed. This assay is a sensitive tool to simultaneously detect 111 cytokines and soluble proteins differently expressed between samples. None of the interleukins, cytokines, and chemokines were detected in the samples obtained from the non-infected and infected brains. Infection with influenza viruses deregulated synthesis of proteins that are involved in the oxidation burst, degranulation of neutrophils, metabolic changes in dendritic cells, recruitment of granulocytes into the brain, neuroinflammation responses, proliferation, and differentiation of neurons, etc. All viruses decreased the synthesis of CX3CL1/Fractalkine, Coagulation factor III, and CD105/Endoglin (Figure 8). WSNad virus induced significantly lower levels of CD54/ICAM, IGFBP-2, IGFBP-5, IGFBP-6, chitinase 3-like 1, cystatin C, and LDL R than WSN virus. On the other hand, WSNad induced high expression of MPO.

The expression of CD54/ICAM-1, IGFBP-5, IGFBP-6, chitinase 3-like 1 (CHI3L1), MPO, OPN, and CD105/Endoglin was inhibited in the brains infected with NS80 virus. Adaptation of NS80 virus resulted in increased expression of the proteins ICAM-1, IGFBP-5, CHI3L1, OPN, and Endoglin compared to non-adapted virus. NS80ad virus is more pathogenic than the NS80 virus, and expression of these proteins induced in NS80ad-infected brains is similar to expression in the brains infected with WSNad virus. Thereby, we assume that the expression of these proteins is not regulated with NS1 protein but depends on the pathogenicity of the virus.

Adaptation of the NS80 virus did not influence the expression of MPO, IGFBP-6, and LDL R in infected brains. The expression of the LDL R is very similar in non-infected brains and the brains infected with NS80 and NS80ad. NS80ad induced lower expression of IGFBP-2 protein than NS80 virus. The MPO protein was not detected in the brains infected with the viruses with deleted NS1 protein. Our results assumed that the MPO, IGFBP-2, and LDL R proteins might be regulated by the NS1 protein of influenza viruses.

## 3. Discussion

The virulence and pathogenicity of the influenza viruses can be increased by serial lung-to-lung passages. The A/WSN/33 virus was isolated from the throat washings of an influenza patient in 1933, and it is the first human influenza virus. Since then, this virus has served as a model to study the origin of the 1918–1919 pandemic, influenza virus structure, replication, pathology, spreading, evolution, etc. Unlike other human viruses, the A/WSN/33 strain can replicate in a variety of cultured cells without the addition of trypsin. The unique mechanism of hemagglutinin cleavage was suggested. Adapted WSN viruses can replicate in a range of murine tissues, and by mouse brain passages, it is possible to prepare a virus that can replicate in the brain [25]. Mouse-adapted viruses replicate in the lungs to a higher titer, induce pathological changes in the bronchi, and possess the ability to infect alveolar cells [26]. We identified the adaptive amino acid changes in PB2, PA, and HA proteins of the NS80ad virus. Adaptation of the WSN virus resulted in the mutation of PB2 and M2 proteins. It was shown that mutations in HA and PA proteins enhance virus replication and increase polymerase activity [27]. The mutation D87N in PB2 protein increases ribonucleoprotein polymerase activity and is associated with the adaptation of influenza viruses [28]. Arginine 45 lines the pore of the M2 proton channel and regulates the transport of protons through the ion channel [29]. We assume that mutation R45H might enhance the activity of the M2 protein.

Our results showed that infection with WSN and NS80 viruses deregulated the expression of more than 600 genes in infected lungs, associated mostly with immune response. Interestingly, we found 116 genes deregulated specifically in NS80 virus-infected cells. These genes are involved in the regulation of cellular machinery and apoptosis. It was shown previously that NS1 protein induces G0/G1 cell cycle arrest [30], thus providing favorable conditions for viral protein accumulation and replication. Therefore, we suggest that loss of NS1 protein promotes cell proliferation, causing unfavorable conditions for virus replication, and this is demonstrated with reduced viral titer obtained from the lungs. However, as noted before, mice infected with NS80 and NS80ad viruses were more lethargic and had more bristled fur than mice infected with WSN and WSNad. One of the causes might be the increased cellular proliferation in the absence of the NS1 protein, leading to pathogenic alterations and damage within the cells [31]. Adaptation of the NS80 virus resulted in an increased ability to inhibit the cellular machinery, block apoptosis, and use the cell for its own replication. It is highly interesting that interferon and interleukin signaling pathways were not positively enriched in the case of comparison of NS80 and NS80ad-infected lungs with WSN-infected lungs. Our results confirmed our previous findings that the influenza virus lacking NS1 protein induces stronger interferon and interleukin response in infected cells and is capable of inducing a strong neuroinflammatory response in the lungs [19].

Some dysregulated signaling and metabolic pathways are closely associated with the development of Alzheimer’s disease, Huntington’s disease, Prion disease, Amyotrophic lateral sclerosis, Parkinson’s disease, and neurodegenerative disease. IAVs do not have a direct tropism to the nervous system. However, high viral load is associated with the dissemination to other organs and increased host response [32]. After infection with avian and pandemic influenza viruses, the virus can be isolated from the neuropils, ependymal, Purkinje cells, neurons, and cerebrospinal fluid [33]. Adaptation of the NS80 virus resulted in increased virus invasion of the CNS. It is known that immune response, especially high levels of proinflammatory cytokines in the blood, is associated with brain histopathology based on the absence of virus RNA [34]. It was shown that infection with IAVs can lead to impairments in hippocampal function, and the histopathological changes are detectable at 30 and even 60 days post-infection in C57BL/6 mice [3]. We have detected the most severe damage in the hippocampus after infection with the WSN virus. Adaptation of the WSN virus did not significantly influence the pathogenicity of the virus. On the other hand, adaptation of the NS80 virus resulted in increased replication of the virus in the lungs, and severe histological changes were observed in the area around the hippocampus. Infection with seasonal H3N2 virus can chronically increase microglia reactivity and amyloid-β plaque burden in the hippocampus of infected mice [35]. Activated microglia and acute inflammation in the brain play an important role in the defense against infection.

We have previously shown that the NS80 virus could induce high levels of proinflammatory cytokines and cytokines associated with high pathogenicity of the virus [19]. The result of GO analysis supports the assumption that increased pathogenicity of adapted viruses is closely related to the immune response elicited after infection with these viruses. KEGG pathway analysis helped to better understand the biological function of genes that are up and downregulated after infection with high loads of IAVs. Adapted viruses, as well as viruses with truncated NS1 protein, activated many metabolic and signaling pathways in the lungs differently than wild viruses. Except for the signaling pathways directly involved in the virus clearing in the lungs, the signaling pathways associated with the development of neurodegenerative diseases were also deregulated. A specific link between IAV infection and neurodegenerative disease progression has been established only recently. IAVs are linked with behavioral and psychological impairments as well as with numerous psychiatric (detachment from reality, hallucinations, loss of consciousness, delirium) and neurological complications (seizures, myalgia, neuralgia, headaches, encephalitis, myelitis, meningitis, and GBS, among others) that can develop into severe dementia [36]. The cellular prion proteins (PrPC), tau protein, amyloid α/β, etc., can play an important role in protection against IAV infection [37]. On the other hand, conformational conversion of PrPC into amyloidogenic isoform PrPSc, Tau hyperphosphorylation, and neurotransmitter imbalance might represent possible neurological mechanisms [38,39].

Although we have not detected active viral particles in the brains, transcription of the genes involved in the RIG-I-like receptor signaling pathway was activated in the brains of infected mice. Unlike the activation of the RIG-I-like receptor pathway activated in the lungs, expression of MDA5, NF-κB, and interferons mRNA did not depend on NS1 protein in the brain. Expression of RIG-I, IRF3, and IFN-β m RNA was significantly increased in the brains infected with WSNad. All viruses induced transcription of t-bet, IFN-γ, IL-2, and IL-10 mRNAs that are responsible for the development of the Th1 subpopulation. Adaptation of the WSN virus influenced the expression of IRF4, IL-2, and IL-12 mRNAs. IRF4 is essential for the development of Th2 cells, which secrete IL-4 and IL-10 cytokines inhibiting the Th1 subset. The NS1 protein influenced the expression of GATA3 and IL-12 mRNA. These transcriptional profiles indicate the dominance of the Th1 subset [40]. Transcriptional profiles of the genes CD38, Gpr18, Fpr2 (markers of classically activated macrophages), Arg1, and Egr2 (markers of alternatively activated macrophages) are significantly activated after infection with all viruses. M1 macrophages are involved in the inflammatory response of host defense [41]. Our results showed that Th1/M2 and Th2/M2 immune pathways were activated. Activation could contribute to the histopathological changes in the infected brains.

Cytokine array revealed that infection of IAV deregulated syntheses of chemokines, soluble proteins, and growth factors involved in neuroprotection. All WSN and NS80 viruses decreased the synthesis of CX3CL1/Fractalkine, Coagulation factor III, FGF-1, and CD105/Endoglin and increased expression of Cystatin C. Chemokine CX3CL1, FGF-1, and a subpopulation of CD105^+^ cells are highly expressed in the convalescent phase after infection and have a neuro-regeneration property [42,43,44]. Coagulation factor III plays an important role in the localization of hemorrhage and regulates CSF flow [45]. Lower concentrations of these proteins can result in inefficient protection of the brain, which can promote neurodegeneration. On the other hand, the level of cystatin C correlates with mortality of brain injury patients, including those who suffered ischemic or hemorrhagic stroke [46].

The WSN virus induced high expression of ICAM-1. A strong correlation exists between traumatic brain injury-mediated inflammation and impairment in functional outcomes following brain trauma and concentration of ICAM-1. On the other hand, the deletion or blocking of ICAM-1 resulted in a better outcome in attenuating neuroinflammation [47]. It is interesting that adapted WSN and NS80 viruses induced lower levels of ICAM-1 and are less pathogenic than WSN virus. Compared to other viruses, the NS80 virus was less pathogenic and induced a very low level of ICAM-1.

IAVs influence the concentration of IGFBP-2, IGFBP-5, and IGFBP-6 proteins. The IGFBP proteins are responsible for the regulation of brain mass homeostasis, differentiation of neural stem cells, and proliferation. IGFBP-2 is a crucial CNS growth factor that is responsible for cognition and information processing in the brain [48]. The WSN virus increased the expression of IGFBP proteins significantly more than the adapted WSN virus. On the other hand, the NS80 virus decreased the expression of these proteins. Moreover, the expression of IGFBP-5 protein was not detected in the brains infected with the NS80 virus. Adaptation of the NS80 virus resulted in increased expression of IGFBP-5 and decreased expression of IGFBP-2. The elevated expression of IGFBP-2 and IGFBP-5 in the brain is of both all-cause dementia and Alzheimer’s disease [48,49]. Deregulation of IGFBP-6 is associated with many diseases, including neurodegeneration. Moreover, IGFBP-6 can act as a chemoattractant towards neutrophils, monocytes, and T cells [50]. The effect of elevated levels of IGFBPs on pathogenicity was less established. However, the data about the biological effect of lower levels of these proteins are not known yet.

The lack of NS1 protein completely abolished the expression of MPO. This finding is a coincidence with the decreased level of GATA3 mRNA in the brains infected with the NS80 viruses. It was shown that the putative MPO promoter region could be regulated by binding proteins from the ets family of transcription factors and duplicate GATA-like sites [51]. The MPO was not detected in the brains infected with non-adapted and adapted NS80 viruses. MPO is a key enzyme in inflammatory and degenerative processes. The significant increase in MPO in the brain regions can affect the development of neurodegeneration [52]. Reduced transcription of GATA3 and absence of MPO in the brains infected with NS80 viruses may be beneficial [53]. It was recently shown that cognitive, memory, and proinflammatory activities were improved in the Alzheimer’s disease mouse model (5XFAD) after producing bone marrow deficient MPO [54]. MPO inhibition was accompanied by decreased demyelination and lower inflammatory cell recruitment in the brains of mice with autoimmune encephalomyelitis [55].

A very low level of CHI3L1 was detected in the brains infected with NS80 viruses compared to WSN viruses. The level of CHI3L1 in the brains infected with NS80ad was increased compared to brains infected with non-adapted NS80 virus but still significantly lower than in the brains of mock mice. A recent study showed an association between CHI3L1 expression levels and hippocampal atrophy, an early event in AD progression [56]. Cytokine OPN regulates innate and adaptive immune responses and has been implicated in neuroinflammatory disorders. Both non-adapted and adapted WSN viruses induced high levels of OPN. Analysis of brain tissue of AD patients reveals a strong correlation between high levels of OPN with dementia severity and neuropathology [57]. Low pathogenic NS80 virus induced a very low level of this protein, and the more pathogenic NS80ad virus induced a significantly higher level of OPN than the NS80 virus.

In this report, we have prepared the adapted NS80 virus by lung-to-lung passaging. The NS80ad virus replicated in the cells to high titer as the control WSN virus and became more pathogenic. The NS80ad virus did not replicate, but vRNA was detected in the brain. We performed global gene expression RNA from lungs infected with WSN, NS80, and NS80-adapted viruses and found the genes deregulated after adaptation of the NS80 virus and the genes deregulated by NS1 protein. Adaptation of the NS80ad virus resulted in increased ability of the virus to manipulate cellular machinery and use the cell for its own replication. Viruses with truncated NS1 protein induced stronger interferon and interleukin responses in infected cells and were capable of inducing strong neuroinflammatory responses in the lungs. KEGG pathway analysis showed that enrichment of these genes was closely associated with neurodegeneration. The transcription of the genes involved in the RIG-I-like receptor pathway and the genes involved in the activation of Th1/M1 and Th2/M2 were activated in the brains of IAV-infected brains. In this case, the NS1 protein did not influence the activity of these genes. Lethal infection with IAVs decreased the expression of proteins responsible for brain regeneration and protection (CX3CL1/Fractalkine, Coagulation factor III, and CD105/Endoglin). Infection with WSN viruses significantly increased the expression of proteins associated with CD54/ICAM-1, IGFBP-2, IGFBP-5, IGFBP-6, CHI3L1, MPO, OPN, cystatin C, and LDL R. The proteins CD54/ICAM-1, IGFBP-5, and MPO were not detected and proteins IGFBP-6, CHI3L1, OPN, CD105/Endoglin, and LDL R were significantly decreased in the brains infected with NS80 and NS80ad viruses. It can be suggested that inflammation is not the only causative of virus pathogenicity in the brains. However, we showed that the influenza viruses regulated the expression of proteins that were responsible for the protection of the brain against infection. This may occur mainly through the NS1 protein. Further investigation is needed to reveal the exact mechanism.

## 4. Materials and Methods

### 4.1. Cells and Viruses

Vero cells (ATCC CCL81) were grown in Dulbecco’s modified Eagle’s medium (Lonza, Verviers, Belgium) containing 10% bovine serum (HyClone Laboratories, Pasching, Austria). The viruses A/WSN/33 (WSN) and the virus lacking the effector and C-terminal domains of NS1 protein (NS80) were prepared by using the reverse genetics system, as previously described [24]. Both viruses were propagated in Vero cells.

### 4.2. Adaptation of WSN and NS80 Viruses in Mice

Female BALB/c mice (4–6 weeks old; body weight approximately 20 g) were purchased from the Institute of Experimental Pharmacology and Toxicology and Animal Breeding at Dobrá Voda (Slovakia). Groups of mice were anesthetized with Zoletil (5 mg/kg) and infected intranasally with 40 µL of supernatant containing WSN or NS80 virus (10^3^ PFU). On the third day post-inoculation, mice were sacrificed by cervical dislocation, and the lungs were harvested and homogenized; 40 µL of supernatant from the centrifuged homogenate was used as inoculum for the next passage. After a total of 5 passages, the virus present in the lung homogenate was passaged into the Vero cells to prepare a virus stock. The adapted viruses were sequenced to confirm the presence of wanted mutations.

To determine the 100% mouse lethal dose (LD100) of non-adapted (WSN and NS80) and adapted (WSNad and NS80ad) viruses, 10-fold serial dilution of each virus was performed, and groups of mice (n = 2) were infected intranasally with each dilution under anesthesia, as described above. The mice were monitored daily for weight loss and survival. The criteria for euthanasia were established by using the total score for observation of possible animal distress. Weight loss exceeding 25% of the original body weight, decrease in appetite, weakness, shivering, depression, and moribund state of animals were monitored twice per day.

### 4.3. Mouse Experiments

To evaluate the virus virulence of the mouse-adapted viruses, groups of mice (n = 3) were inoculated intranasally with lethal doses of viruses in 40 µL or mock inoculated with PBS to serve as controls. Mice were sacrificed by cervical dislocation on the 3rd day p.i., and the lungs were perfused with 100 mL of cold PBS. The lungs and brains were aseptically harvested and homogenized in 0.5 mL of PBS, and the aliquots from the organ homogenate were stored at −80 °C.

### 4.4. Histological Analysis

The collected brains were processed according to standard histology procedures, being fixed in 10% neutral-buffered formalin and embedded in paraffin. Five-micrometer sections were stained with hematoxylin (Sigma-Aldrich, St. Louis, MO, USA) and eosin (Sigma-Aldrich, Burlington, MA, USA) and examined by light microscopy.

### 4.5. Viral Growth Kinetics and Determination of the Virus Titer

The ability of adapted viruses to grow in Vero cells was analyzed by multiple-cycle growth kinetics. Confluent Vero cells were infected with WSN, WSNad, NS80, and NS80ad at a multiplicity of infection (MOI) of 0.01, overlaid with serum-free DMEM and incubated at 37 °C. Cell supernatants were harvested at 24, 48, and 72 h p.i.

The supernatants from the cells, infected lungs, and brains were used for virus quantification. The viral titers were determined as TCID50 titers by the endpoint dilution assay in Vero cells. The titers were calculated by the Reed–Muench method.

### 4.6. Semi-Quantitative Reverse Transcription-Polymerase Chain Reaction (RT-PCR)

Total RNA from the brain homogenates was extracted using the SV Total RNA Isolation System (Promega, Madison, WI, USA). A total of 400 ng/µL RNA was reverse-transcribed using random hexanucleotide primers and the MuLV reverse transcriptase (Finnzyme; Thermo Fisher Scientific, Vilnius, Lithuania). The primers targeting β-actin, RIG-I, MDA-5, NF-κB, IRF3, IRF7, IRF4, IFN-α, IFN-β, IFN-ε, IFN-γ, IFN-λ, t-bet, IL-2, IL-4, IL-10, GATA, CD38, Gpr18, Frp2, Arg1, and Egr2 were previously described [19,23,24,58,59,60]. The band intensity of the PCR products was determined using ImageJ software version 2 (Syngene). β-actin was used as an internal control to normalize the expression of the mRNA levels between different samples.

### 4.7. Western Blot

The Western blot was performed as previously described [23]. Briefly, the brain homogenates were lysed in extraction buffer (1% Triton X-100, 1 mM EDTA, 20 mM Tris/HCl, pH 7.4) containing 2 mM Sodium fluoride, 1 mM Sodium orthovanadate, 1 mM Phenylmethylsulphonyl fluoride, 1 μg/mL Aprotinin, 1 μg/mL Leupeptin, 10 μg/mL β-Glycerophosphate, and 1 μg/mL Pepstatin. The concentration of proteins was determined using the Pierce BCA Protein assay kit (Thermo Fisher Scientific, Rockford, IL, USA). The same amount of protein per sample was run using Laemmle loading buffer in 12.5% SDS-polyacrylamide gels under reducing conditions and then transferred to a polyvinylidene difluoride (PVDF) membrane (Bio Rad, Hercules, CA, USA). The proteins were detected with IRF3 polyclonal antibody (PA5-87506), Phospho-ITF3 (Ser385) polyclonal antibody (PA5-36775), IRF7 polyclonal antibody (PA5-20281), Phospho-IRF7 (Ser471, Ser472) polyclonal antibody (PA5-114592) (Invitrogen, Rockford, IL, USA), and HSC-10 (SC-7298) (Santa Cruse Biotechnology, Inc., Heidelberg, Germany). After washing in PBS with Tween-20 (Sigma Aldrich), the membranes were incubated with the Protein A–horseradish peroxidase conjugate, and the antibody–protein complexes were detected using the chemiluminescence (ECL) reagent (Amersham, Thermo Fisher Scientific, Rockford, IL, USA). The band intensity was determined using ImageJ software (Syngene).

### 4.8. RNA Sequencing and Gene Expression Analysis

RNA sequencing via Illumina platforms, based on the mechanism of SBS (sequencing by synthesis) and standard bioinformatic analysis, was performed by Novogene Company, Ltd., Cambridge, (UK). Original data files from high-throughput sequencing platforms were transformed into sequencing reads by CASAVA base recognition. The raw reads were filtered to remove (i) reads containing adaptors, (ii) reads containing N > 10%, and (iii) reads Qscore of over 50% bases below 5. The clean reads were mapped to the mouse reference genome using Hierarchical Indexing for Spliced Alignment of Transcripts 2 (HISAT2). The DEG analyses were performed with DEseq version 1.20.0 following the parameter of fold change > 2 and *p*-value ˂ 0.05. The gene expression level was estimated by FPKM (short for the expected number of Fragments Per Kilobase of transcript sequence per million base pairs sequenced). ClusterProfiler version 3.8.1 software and WebGestalt were used for enrichment analysis, including GO enrichment, KEGG, and Panther and Reactome database pathway analyses [61,62]. Hierarchical clustering of FPKM values was performed using Cluster 3.0, and heat maps were visualized in Java TreeView 3.0 [63,64].

### 4.9. Identification of Somatic Mutations

The sequencing data obtained from RNA-seq were uploaded to the Galaxy web platform, and we used the server at usegalaxy.eu to analyze the data [65]. The quality of reads was analyzed using FastQC (v 073 + galaxy0). Trimming of adapters and quality trimming was performed using Trim Galore! (v 0.6.7 + galaxy0) and Trimmomatic (v 0.39 + galaxy0). Reads were aligned to reference sequences using BWA-MEM (v 0.7.17.1). The mate coordinates were filled in the bam files using samtools fixmate (v 1.13). Duplicates were marked using the markdup option of samtools (v 1.13 + galaxy1). Single nucleotide polymorphisms (SNPs) of viral genes were called using FreeBayes (v 1.1.0.46-0), and the output was set to vcf. SnpSift filter (v 3.4), which was utilized to keep variants that were covered at least 11 times and have a quality score above 30. SNPs were vizualized using Integrative Genomics Viewer (IGV) [66].

### 4.10. Cytokine Array

Brain tissue homogenates (100 µL) were lysed, and the protein concentration was determined using the Pierce BCA Protein assay kit (Thermo Fisher Scientific, Rockford, IL, USA). Chemokine expression in the cell lysates was assessed using the Proteome Profiler Mouse XL Cytokine Array Kit (R&D Systems, Minneapolis, MN, USA). Signal intensities on autoradiography films were quantified using ImageJ software version 2 (Syngene). The expression levels of cytokines were normalized to the expression level of reference spots. The assay was performed in duplicate to ensure reproducibility of the results.

### 4.11. Gene Set Enrichment Analysis (GSEA)

All FPKM values for lungs infected with WSN, NS80, and NS80ad virus and controls (NC) were converted to the GCT expression dataset. Ontology gene sets were downloaded from Broad Institute’s Molecular Signatures Database. Gene Set Enrichment Analysis (v4.3.2) was used to test the relationship between RNA-seq expression data and the Ontology gene sets. Gene sets enriched in less than 15 genes, and more than 500 genes were excluded from the analysis. Gene sets with a false discovery rate (FDR) value < 0.25 and *p* < 0.05 after performing 1000 permutations were considered to be significantly enriched.

### 4.12. Statistical Analysis

Statistical analysis was performed by comparing the control group (non-infected mice—Mock) to infected mice or WSN-infected mice to NS80-infected mice, respectively. Data were analyzed using the unpaired Student’s *t*-test for data with two groups and ANOVA followed by Tukey’s post hoc test. *p* < 0.05 was statistically significant.

## 5. Conclusions

Truncated NS1 protein in the NS80 virus promoted cell proliferation, causing unfavorable conditions for virus replication, and this is demonstrated with reduced viral titer obtained from lungs in comparison to WSN and adapted viruses. Adaptation of attenuated virus with truncated NS1 protein (NS80) resulted in increased replication in the lungs of infected mice, most likely due to increased ability to inhibit the cellular machinery, block apoptosis, and use the cell for its own replication. Adapted influenza viruses with truncated NS1 protein induced strong interferon and interleukin responses in infected cells and were capable of inducing strong neuroinflammatory responses in the lungs. Transcriptome analysis of the gene expression in the lungs revealed that infection with wild-type WSN and NS1 truncated viruses resulted in different regulation of genes involved in signaling pathways associated with cell proliferation, inflammatory response, and development of neurodegenerative diseases. NS1 protein did not influence the genes involved in the RIG-I-like receptor signaling pathway in the brains. Transcription of GATA3 mRNA and expression of MPO, IGFBP-2, CHI3L1, and LDL-R proteins was regulated in the brain by NS1 protein. In conclusion, we propose that infection with the influenza virus might influence the expression of proteins involved in brain function, and this might occur mostly through the NS1 protein.

## Data Availability

The data supporting the results are available from the corresponding author upon reasonable request.

## Supplementary Materials

The following supporting information can be downloaded at: https://www.mdpi.com/article/10.3390/ijms25052460/s1.
