# Supplementary material for: Changes in the Expression of Proteins Associated with Neurodegeneration in the Brains of Mice after Infection with Influenza A Virus with Wild Type and Truncated NS1
Source: Int J Mol Sci. 2024 Feb 20;25(5):2460. doi: 10.3390/ijms25052460 (PMC10931352; doi:10.3390/ijms25052460)
Supplement: Supplementary file 1 [file ijms-25-02460-s001.zip › ijms-2857144-supplementary.pdf]

.

Sample name	Raw reads	Clean Reads	Total Map		Proper Map	
Mock-1	45332750	44743638	39590302	**88.48%**	36178764	**80.86%**
Mock-2	81332450	80048738	69686755	**87.06%**	58175438	**72.68%**
WSN	39496130	39074648	36448824	**93.28%**	33729024	**86.32%**
WSN	45434642	44840648	41708707	**93.02%**	37800180	**84.3%**
WSN	44262588	43624318	40572579	**93.00%**	37350580	**85.62%**
WSN ad	39348744	38937330	36336954	**93.32%**	33220074	**85.32%**
NS80	45552098	44983290	41681965	**92.66%**	38507578	**85.6%**
NS80	40565552	40076928	36917427	**92.12%**	34146938	**85.2%**
NS80	51294628	40739584	36615148	**89.88%**	33468954	**82.15%**
NS80 ad	42170062	41539672	38674653	**93.1%**	35616364	**85.74%**
NS80 ad	50142384	49519296	45826736	**92.54%**	42299136	**85.42%**
NS80 ad	43139690	42753362	39745264	**92.96%**	36903794	**86.32%**

Table S1. Summary of data obtained from the sequenced RNA-seq libraries.

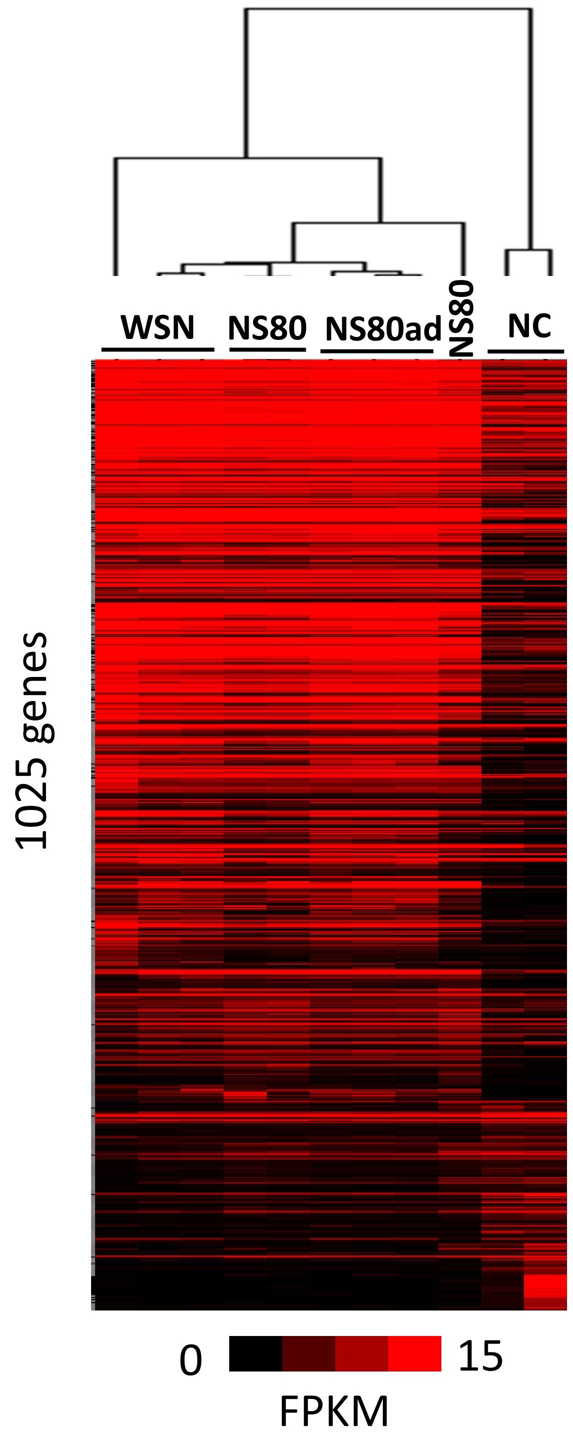

Figure S1. Hieratical clustering of FPKM values from lungs infected with WSN, NS80, NS80ad and uninfected control (NC). Selection of 1025 genes was done by comparing expression value between WSN and NS80 infected cells relative to uninfected controls (Figure 2A).

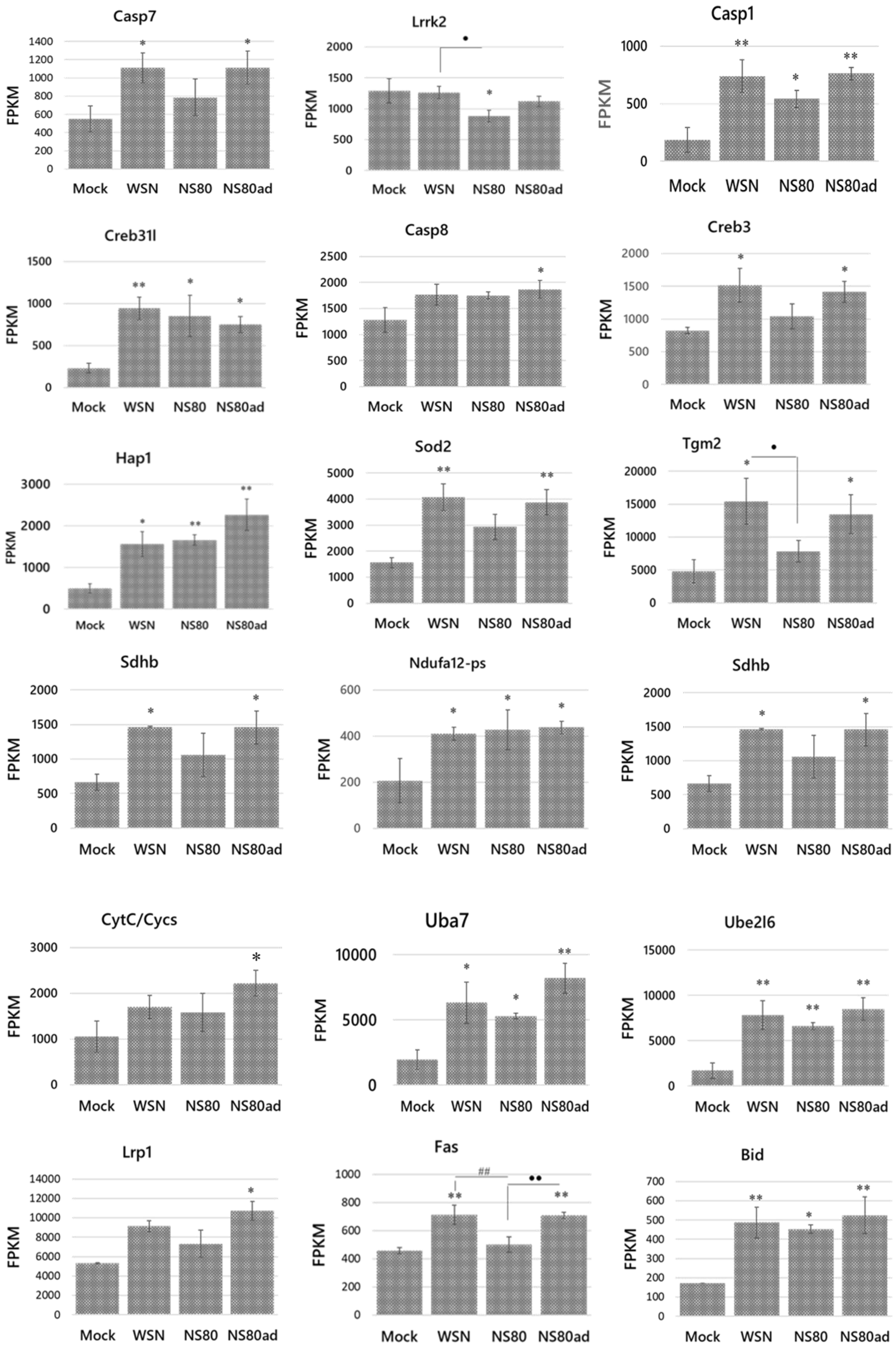

Casp7
Lrrk2
Casp1
Creb31l
Casp8
Creb3
Hap1
Sod2
Tgm2
Sdhb
Ndufa12-ps
Sdhb
CytC/Cycs
Uba7
Ube2l6
Lrp1
Fas
Bid
FPKM
Mock
WSN
NS80
NS80ad

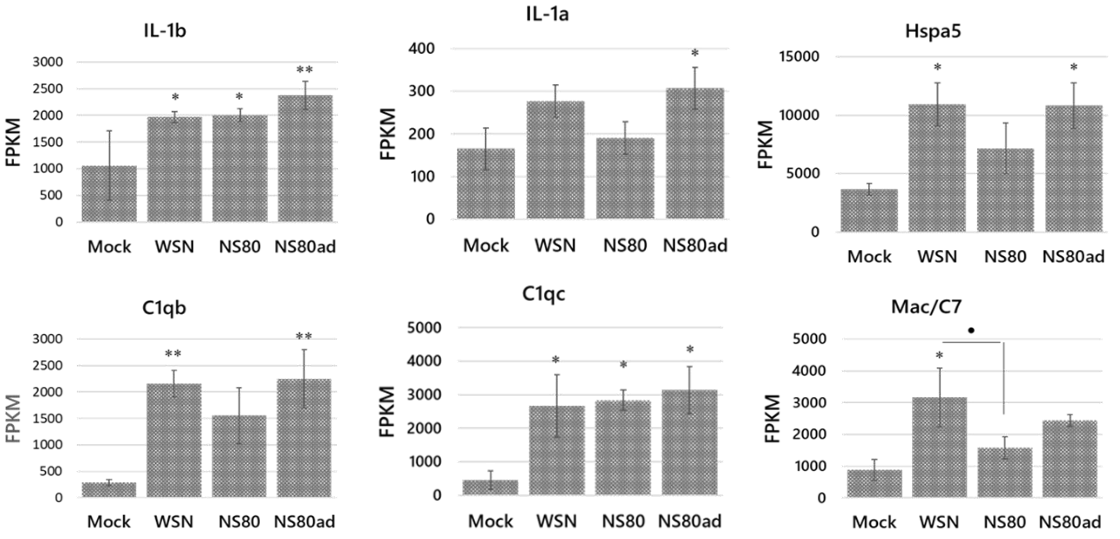
IL-1b
3000
2500
2000
1500
1000
500
0
FPKM
Mock WSN NS80 NS80ad
IL-1a
400
300
200
100
0
FPKM
Mock WSN NS80 NS80ad
Hspa5
15000
10000
5000
0
FPKM
Mock WSN NS80 NS80ad
C1qb
3000
2500
2000
1500
1000
500
0
FPKM
Mock WSN NS80 NS80ad
C1qc
5000
4000
3000
2000
1000
0
FPKM
Mock WSN NS80 NS80ad
Mac/C7
5000
4000
3000
2000
1000
0
FPKM
Mock WSN NS80 NS80ad

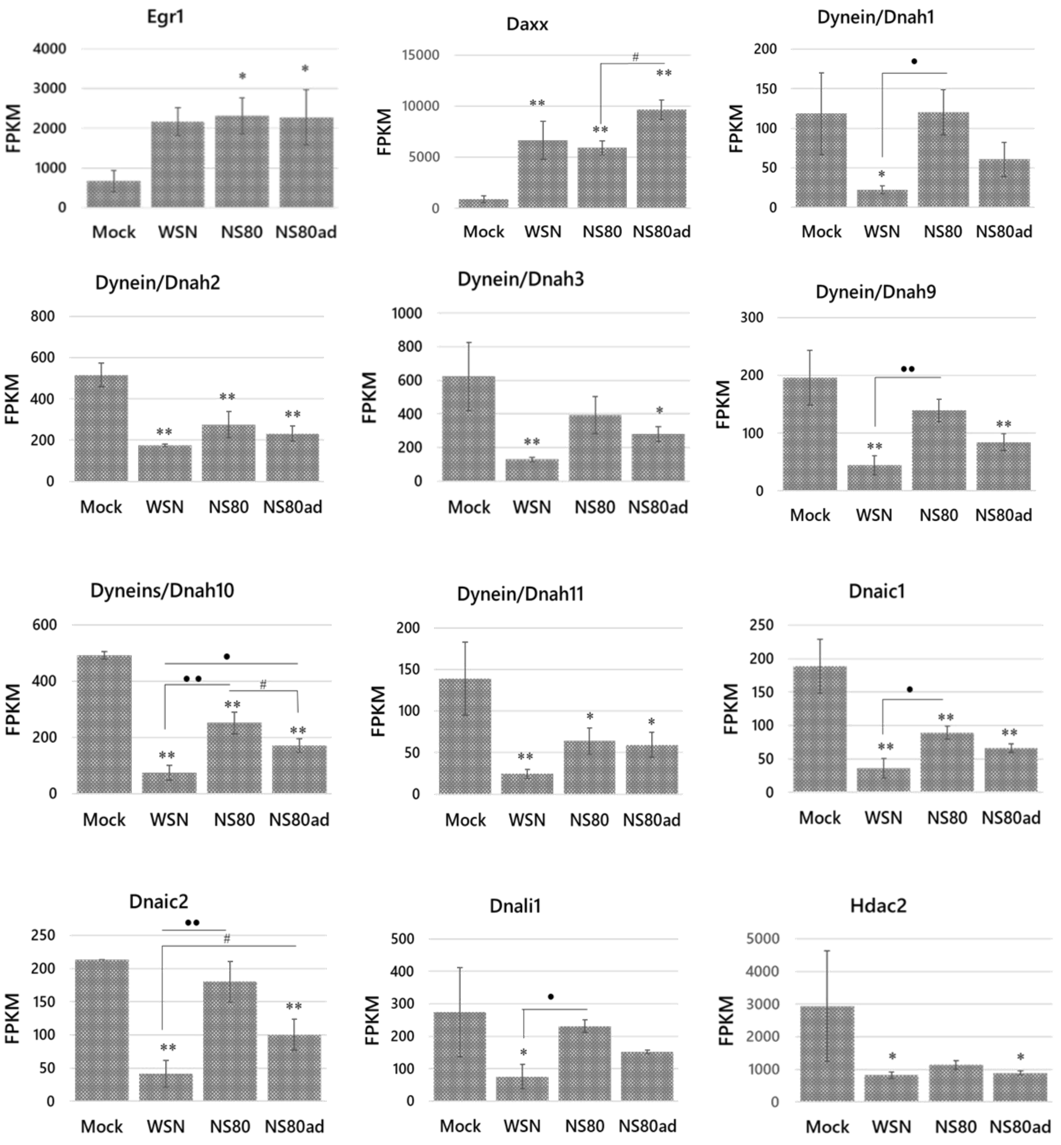
Egr1
4000
3000
2000
1000
0
FPKM
Mock WSN NS80 NS80ad
Daxx
15000
10000
5000
0
FPKM
Mock WSN NS80 NS80ad
Dynein/Dnah1
200
150
100
50
0
FPKM
Mock WSN NS80 NS80ad
Dynein/Dnah2
800
600
400
200
0
FPKM
Mock WSN NS80 NS80ad
Dynein/Dnah3
1000
800
600
400
200
0
FPKM
Mock WSN NS80 NS80ad
Dynein/Dnah9
300
200
100
0
FPKM
Mock WSN NS80 NS80ad
Dyneins/Dnah10
600
400
200
0
FPKM
Mock WSN NS80 NS80ad
Dynein/Dnah11
200
150
100
50
0
FPKM
Mock WSN NS80 NS80ad
Dnaic1
250
200
150
100
50
0
FPKM
Mock WSN NS80 NS80ad
Dnaic2
250
200
150
100
50
0
FPKM
Mock WSN NS80 NS80ad
Dnali1
500
400
300
200
100
0
FPKM
Mock WSN NS80 NS80ad
Hdac2
5000
4000
3000
2000
1000
0
FPKM
Mock WSN NS80 NS80ad

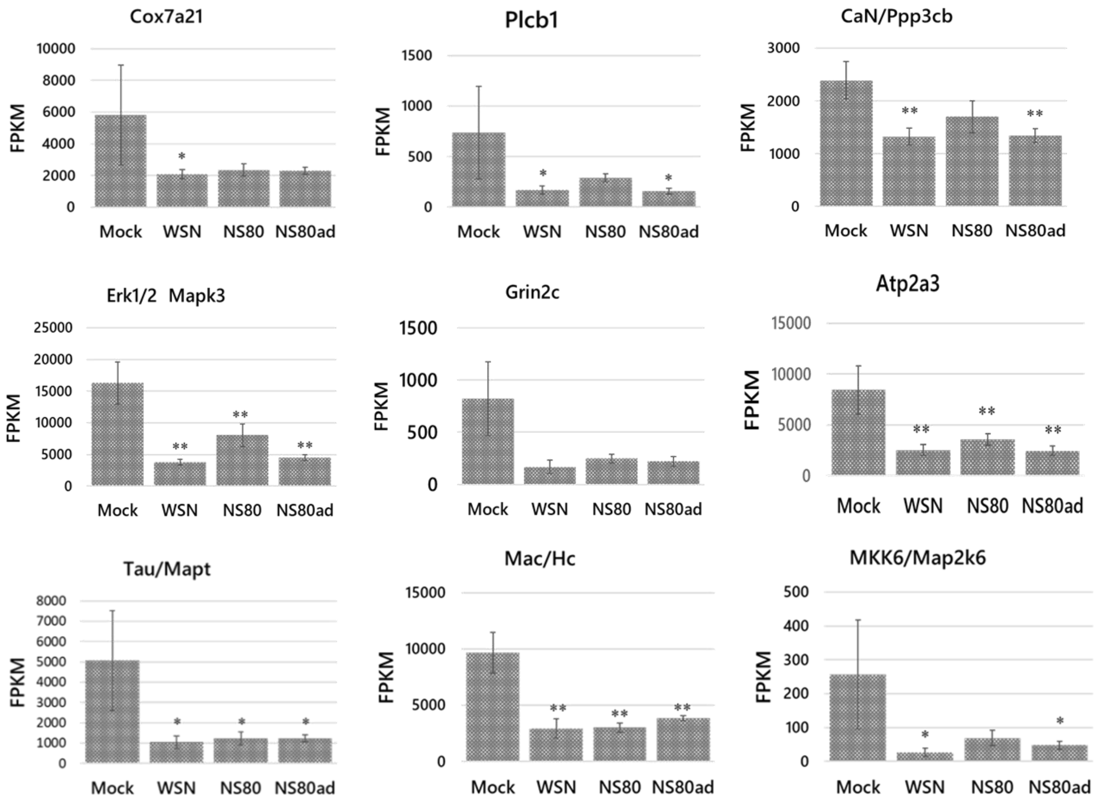

Figure S2. The FPKM values obtained from RNA seq data analyses from brains (n=3) infected with WSN (n=3), NS80 (n=3), and NS80ad(n=3). Data are presented as the mean ± SD. Data were statistically evaluated using one-way ANOVA and post hoc Tukey's HSD test; #,•,* $p < 0.05$; ••,** $p < 0.01$; *** $p < 0.001$.
